# Investigating Suicide Risk Factors Among Appalachian West Virginian Adults

**DOI:** 10.13023/jah.0604.05

**Published:** 2025-01-29

**Authors:** Erin D. Caswell, Angela M. Dyer, Summer D. Hartley, Caroline P. Groth, Mary Christensen, Sahiti K. Tulabandu, Bryce K. Weaver, Ruchi Bhandari

**Affiliations:** West Virginia University; Hartley Health Solutions; Hartley Health Solutions; West Virginia University

**Keywords:** Appalachia, Appalachian health, mental health services, rural-urban disparities, social determinants of health, suicide

## Abstract

**Introduction:**

Suicide rates in the United States have increased over the past two decades, with rural areas, particularly the Appalachian Region, facing unique challenges that elevate suicide risk. These include economic hardships, social isolation, and limited access to mental health services.

**Purpose:**

This study addresses critical gaps in understanding lifetime suicide risk in West Virginia (WV), a predominantly rural state entirely within the Appalachian Region. By identifying the factors driving urban-rural differences in suicide risk, this research seeks to inform interventions tailored to the state's distinct needs and provide insights applicable to the broader Appalachian

**Region Methods:**

Using 2021 Mountain State Assessment of Trends in Community Health (MATCH) survey data, we examined socioeconomic and related factors associated with lifetime suicide risk in WV, measured by the first item of the Suicide Behaviors Questionnaire-Revised (SBQ-R). Logistic regression models identified significant risk and protective factors. Models were stratified by rural residence using 2023 Rural-Urban Continuum Codes (RUCC) to examine rural-urban disparities, given WV’s predominantly rural yet urban-diverse geography.

**Results:**

In the weighted sample (N=372,665), 27.5% reported lifetime suicide risk. Those with suicidal thoughts were younger (median age 41), unmarried, in poorer health, and often enrolled in Medicaid. Despite WV’s rural profile, 60.21% of respondents resided in urban-classified counties. Rural residents showed lower odds of suicidal thoughts or behaviors (aOR = 0.87), but factors such as substance use (aOR = 3.75), unmarried status (aOR = 1.51), and mental health disorders (aOR = 2.93) were significant risk factors.

**Implications:**

Suicide risk factors in WV differ from broader suicidology findings, underscoring the need to address substance use, chronic pain, and mental health in prevention strategies. Further research is needed to explore regional differences in the Appalachian Region for better-targeted interventions.

## INTRODUCTION

Suicide is a critical public health issue in the United States (U.S.), with rates rising over the past 20 years.^1^ From 2001 to 2021, U.S. suicide rates increased by 32%.^2^ In 2021, 12.3 million adults reported serious suicidal thoughts, with 3.5 million making plans and 1.7 million attempting suicide.^3^ Suicide is a complex issue influenced by socioeconomic and structural determinants like income, geographic location, and healthcare access.^4^–^7^ For instance, suicide risk is higher among individuals living below the federal poverty level,^4^, ^8^ lacking healthcare access,^9^ using substances^10^ and living in rural areas.^6^ In rural Appalachia, economic hardships, social isolation, and disrupted healthcare services have further increased suicide risk factors.^11^, ^12^

The Appalachian Region faces the challenges of lower educational attainment, limited access to mental health services, higher poverty rates, and an increased prevalence of chronic pain.^13^ Increased substance use compared to other U.S. rural areas particularly exacerbate these issues.^14^ Rural Appalachia has experienced disproportionately high rates of alcohol-related deaths, prescription opioid misuse, overdose deaths, and suicide).^15^ In 2022, the regional suicide rate reached 22.7 per 100,000, which was substantially higher than the 2022 national average of 14.2 per 100,000.^16^, ^17^ In West Virginia (WV), the only state entirely within Appalachia, the suicide rate is among the highest in the region, with over 50% of the population living in rural areas.^18^ While WV has urban areas, they often share characteristics with rural areas, such as limited healthcare access, economic hardship, and lower population density.^19^ This unique context allows for a focused study on rurality, regional culture, and suicide risk, potentially offering insights for addressing suicide in other parts of Appalachia.

**Figure 1:**
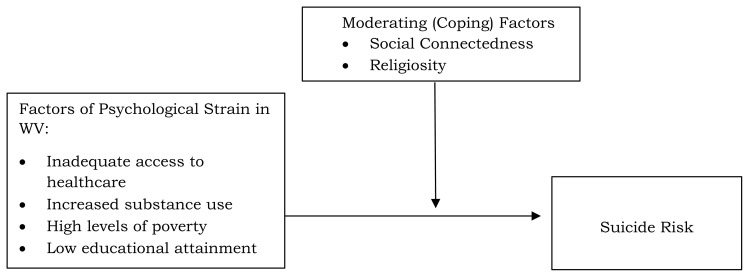
Contextualizing Lifetime Suicide Risk in West Virginia Using the Strain Theory of Suicide

The strain theory of suicide provides a critical framework for comprehending the dynamics between socioeconomic disparities and lifetime suicide risk, which is particularly relevant in WV **(**Fig 1). According to this theory, individuals may experience heightened suicide risk when psychological strain, arising from unresolved or conflicting life stressors (such as those associated with socioeconomic disparities) exceeds their coping mechanisms.^20^ The assessment of lifetime suicide risk is especially important in population-based research, as it reflects the cumulative effects of long-term socioeconomic and psychological strain, aligning with strain theory by demonstrating how chronic stressors gradually erode coping capacities over time.^21^, ^22^ In rural Appalachia, strains such as economic hardship, chronic pain, and limited access to healthcare can deepen feelings of hopelessness and despair, further intensifying the imbalance between strain and coping.^23^ In the context of WV and similar rural Appalachian areas, protective factors like social connectedness and religiosity might mitigate the effects of psychological strains by serving as coping mechanisms, underscoring their importance in these high-risk regions.^24^ Thus, the strain theory emphasizes the need to assess lifetime suicide risk in population-based research, considering the cumulative effects of various life strains on mental health and suicide risk.^21^, ^22^

However, research on lifetime suicide risk in WV remains limited, and existing research efforts have been limited in scope and efficacy. For example, surveillance mechanisms have been unable to capture the full extent of suicide-related data, particularly in rural areas, hampering both prevention strategies and resource allocation.^21^, ^25^, ^26^ The challenges in capturing these data include geographical barriers, limited healthcare infrastructure, and social stigma surrounding mental health issues, which often result in underreporting of suicide cases and related behaviors.^27^ Consequently, these data gaps persist in our understanding of the factors driving suicide rates in WV, impeding the development of targeted interventions tailored to the state’s specific needs.^5^, ^28^, ^29^ Additional suicide research in WV is crucial for identifying at-risk populations, understanding the potential underlying causes, and developing effective prevention and intervention strategies.

To address the need for comprehensive suicide research in WV, this cross-sectional study aims to explore socioeconomic and related factors of lifetime suicide risk in WV and identify urban-rural disparities using data collected from the 2021 Mountain State Assessment of Trends in Community Health (MATCH) survey, a public health surveillance system in the state.^30^ Given the known association between rurality and lifetime suicide risk, we hypothesize that urban and rural areas within WV will show significant differences in suicide risk factors. This research is essential for developing evidence-based interventions to reduce suicide rates in these areas and may provide a model for addressing suicide in other Appalachian regions.

## METHODS

### Population Sample and Study Design

The MATCH survey is a state-based population health monitoring system designed to capture various health indicators at the state, region, and county levels in WV. It was created through a collaborative effort between the WV University Health Affairs Institute (Health Affairs) and two state agencies: the WV Department of Health and the WV Department of Human Services. These state agencies were formerly part of the WV Department of Health and Human Resources during the 2021 MATCH survey’s inaugural year.^31^ The 2021 MATCH survey employed a sophisticated dual-frame design, which included an address-based sampling frame to target the general adult WV population and a Medicaid administrative frame to provide an oversample of adult Medicaid recipients. The survey design included additional oversampling strategies, particularly targeting low-income and black or African American populations, to maximize the representation of these subgroups. In the address-based sampling frame, addresses were selected and the adult (aged 18 years or older) at the residence with the most recent birthday was invited to participate. In the Medicaid frame, the adult individual was selected directly. Through random selection, WV adults (aged 18 years or older) living in non-group housing were invited to participate in the survey via a sequential “push-to-web” strategy comprised of up to four mailings to enhance response rates.

WV adults were invited to participate in the survey via computer-assisted web interviewing, paper-and-pencil interviewing, or telephone. Data were collected between Aug. 31, 2021, and Feb. 28, 2022. Of the 88,004 (100%) WV households/individuals selected to participate in the 2021 MATCH survey, 81,073 (92.12%) of these met the eligibility requirements for survey participation. An address selected from the address-based sampling frame was deemed ineligible if it was determined to not represent a household (i.e., vacant). An adult selected from the Medicaid administrative frame was deemed ineligible if they were not a resident of a household (e.g., living in a nursing home). A total of 16,185 survey responses were included in the final analytic dataset and yielded 20% for the overall unit response rate (AAPOR RR2).^32^ The weighting strategy included a three-step approach. First, individual weights for each sampling frame were adjusted for the survey sampling design, eligibility, nonresponse, and in the case of the address-based sample, correction for the number of adults at the address. Second, these weights were adjusted to blend the Medicaid and address-based sampling frames. Finally, a coverage adjustment through calibration ensured the final weights aligned with external population distributions for key characteristics like age group, race, and Medicaid status, optimizing representation at state and substate levels. Detailed information on the 2021 MATCH survey methodology and weighting is published elsewhere.^30^ The final weighted sample for the present study included all WV adult residents (aged 18 years or older) who did not have a missing response to the lifetime suicide risk measure (N=372,665).

### Measures

#### Lifetime Suicide Risk

The outcome of interest for this study was lifetime suicide risk. For evaluating the prevalence of lifetime suicide risk in our population, we used the MATCH survey measure, “Have you ever thought about or attempted to kill yourself?” taken from the first item of the Suicide Behaviors Questionnaire-Revised SBQ-R. 33 While the suicide risk continuum model^34^ suggests that suicidal thoughts and behaviors range from passive thoughts of death to active suicidal ideation, planning, and attempts,^35^ it fails to capture the fluctuating and episodic nature of suicide risk. Assessing lifetime risk considers these variations and provides a more accurate depiction of an individual’s overall risk across their lifespan.^22^ The SBQ-R demonstrates strong internal consistency and structural validity for identifying lifetime suicide risk in the general adult population.^36^ The first item of the SBQ-R has six response options that correspond to a pre-established score: 1 (Never), 2 (It was just a brief passing thought), 3 (I have had a plan at least once to kill myself but did not try to do it), 3 (I have had a plan at least once to kill myself and really wanted to die), 4 (I have attempted to kill myself, but did not want to die), and 4 (I have attempted to kill myself, and really wanted to die). The responses receive the following scores based on the options: 1, 2, 3, 3, 4, and 4, respectively. Lifetime suicide risk was analyzed as a binary variable using a cutoff score of two or greater to indicate the prevalence of lifetime suicide risk.^33^ This approach captures a broad range of suicidal thoughts and behaviors, which may provide a more comprehensive view of lifetime risk across the study population.

#### Socioeconomic and Other Related Factors and Control Variables

In this study, the primary independent variables were socioeconomic and related factors, which included education (less than a high school education, high school education or higher), Federal Poverty Level (FPL) categories (≤100%, 100.01%–199.99%, and ≤200%), insurance coverage (none, other, Medicaid/Medicare), self-reported fair or poor health (yes, no), combined past 12 month substance use and past month heavy drinking (substance use and heavy drinking, substance use or heavy drinking, no substance use or heavy drinking) depression, anxiety, or Post-Traumatic Stress Disorder (PTSD) (yes, no) and chronic pain (yes, no). Consistent with suicidology literature, control variables included age, biological sex, race, marital status (measured as a binary yes/no to married or cohabitating), and metropolitan status (rural v. non-rural).^4^, ^8^, ^37^ Metropolitan status was based on the 2023 Rural-Urban Continuum Codes (RUCC)^38^ and we reported age both as a categorical and continuous variable to better describe our study population. To increase statistical power, the continuous age variable was used for the final analyses.

### Statistical Analysis

All analyses were conducted using SAS® 9.4.^39^ Cluster size, strata, and sample weights accounted for the MATCH survey’s complex sampling design, where clusters represent primary sampling units (PSUs) corresponding to households identified by their addresses in the stratified sampling frame. Descriptive statistics provided sample distribution for the total population and by lifetime suicide risk group. Raw frequencies and percentages representing weighted proportions were reported. Rao-Scott χ^2^ tested for subgroup differences among categorical variables and a pooled variance t-test was used for the continuous age variable. Bivariate logistic regression was used to examine the associations between individual variables and lifetime suicide risk. Multicollinearity was tested by examining variance inflation factors and was determined to be absent. The final multivariable logistic regression model adjusted for education, poverty, insurance coverage, fair or poor health, substance use, chronic pain in the past 12 months as told by a doctor, depression, anxiety, or PTSD in the past 12-months as told by a doctor, and rural residence. Adjusted odds ratios (aOR) were reported. Additionally, due to a significant interaction between rural residence and several other predictor variables, the final adjusted model was stratified by rurality, as defined by the 2023 RUCC.^38^ Lastly, a non-response analysis was conducted to evaluate the difference between respondents and non-respondents to the SBQ-R, since evidence suggests that refusal to answer suicide screening questions may characterize individuals at a higher risk of suicide.^40^, ^41^ The response event (response v. missing response) was modeled to assess potential biases arising from individuals refusing to answer or providing incomplete responses to the suicide risk question.

## RESULTS

### Descriptive Statistics

Our study utilized lifetime suicide risk as measured by the SBQ-R to understand the prevalence and factors associated with suicide in WV. The final study sample consisted of 15,660 respondents, representing a weighted total of 372,665 West Virginians. The sample encompassed individuals aged 18 years or older, with the median age being 50.17 (IQR=30.02) years. Most of the sample identified as white (93.62%), held a high school education or higher (87.60%) and were enrolled in some type of health insurance (90.45%). Three-fourths (75.99%) of participants rated their health better than “fair” or “poor.” Many participants reported being told by a doctor that they have depression, anxiety, or PTSD (43.02%), reported substance use in the past 12 months or heavy drinking in the past 30 days (40.39%), and reported being told by a doctor that they have chronic pain (32.17%). Additionally in the weighted sample, a majority of participants lived in a rural area (39.79%), were married or living with a partner (54.29%), and fell within the ≥200% FPL category (52.73%). See Table 1 for complete descriptive statistics of the final study sample. The final study sample was also stratified by lifetime suicide risk **(**Table 1). More than a quarter (27.50%) of WV adults indicated lifetime suicide risk. Participants who reported lifetime suicide risk tended to be younger, with a median age of 40.99 (IQR=26.08) years. Of those who reported lifetime suicide risk, more than half (53.82%) were not married or cohabitating. Finally, a large proportion of those reporting lifetime suicide risk fell within the ≤100% FPL category (29.55%), rated their health as “fair or poor,” (29.89%), and were enrolled in Medicaid (41.12%).

### Bivariate and Multivariable Results

Table 2 presents bivariate and multivariable associations between sociodemographic characteristics and the outcome variable of interest, lifetime suicide risk. Age showed a significant inverse association with lifetime suicide risk, with each one-unit decrease in age corresponding to a 3% increase in odds of lifetime suicide risk in both bivariate (OR = 0.97, 95% CI: 0.97–0.97) and multivariable (aOR = 0.97, 95% CI: 0.97–0.98) analyses. Additionally, marital status, chronic pain, depression, anxiety, PTSD, substance use, and rurality were significant predictors of lifetime suicide risk in both analyses. In the bivariate analyses, those who were not married or cohabitating had 61% higher odds of lifetime suicide risk compared to married or cohabitating individuals (OR = 1.61, 95% CI: 1.44–1.80). This association remained significant in the multivariable analyses, although, for those who were not married or cohabitating, the odds of lifetime suicide risk were now 35% higher (aOR = 1.35, 95% CI: 1.19–1.54). Compared to those without chronic pain, those with chronic pain had higher odds of lifetime suicide risk in both bivariate (OR = 1.67, 95% CI: 1.48–1.89) and multivariable (aOR = 1.40, 95% CI: 1.20–1.64) analyses. Participants with depression, anxiety, or PTSD showed increased odds of lifetime suicide risk in both bivariate (OR = 3.72, 95% CI: 3.29–4.19) and multivariable (aOR = 2.65, 95% CI: 2.30–3.06) analyses, compared to those who did not report a mental health condition. Participants who had used substances in the past 12 months or reported heavy drinking in the past 30 days had elevated odds of lifetime suicide risk than those who did not in both bivariate (OR = 3.31, 95% CI: 2.47–4.44) and multivariable (aOR = 2.37, 95% CI: 1.70–3.30) analyses. Contrary to previous findings and study hypotheses, living in a rural area versus non-rural had significantly lower odds of lifetime suicide risk in both the bivariate (OR = 0.83, 95% CI: 0.75–0.93) and multivariable (aOR = 0.87, 95% CI: 0.77–0.98) models.

### Multivariable Results Stratified by Rurality

Due to significant interactions between rural residence and several predictor variables, the final multivariable logistic regression model was stratified by rurality **(**Table 3). In rural and non-rural areas, younger age was significantly associated with an increased lifetime suicide risk for rural residents (aOR = 0.98, 95% CI: 0.97–0.98) and non-rural residents (aOR = 0.97, 95% CI: 0.97–0.98). Individuals with self-reported fair or poor health, compared to those with better self-reported health, had significantly higher odds of lifetime suicide risk in both rural (aOR = 1.47, 95% CI: 1.18–1.82) and non-rural areas (aOR = 1.53, 95% CI: 1.21–1.91). On the other hand, those with less than a high school education had a significantly lower lifetime suicide risk compared to those with a high school education or higher in rural (aOR = 0.56, 95% CI: 0.42–0.75) and in non-rural areas (aOR = 0.64, 95% CI: 0.46–0.88). The odds of lifetime suicide risk were notably higher among rural residents who had used substances in the past 12 months and reported heavy drinking in the past 30 days compared to those who did not (aOR = 3.75, 95% CI: 2.24–6.28). Similarly, rural residents with chronic pain (aOR = 1.54, 95% CI: 1.25–1.89), those who were unmarried or not cohabitating (aOR = 1.51, 95% CI: 1.26–1.81), and individuals with depression, anxiety, or PTSD (aOR = 2.93, 95% CI: 2.41–3.57) also had significantly higher odds of lifetime suicide risk compared to their counterparts without these characteristics. Additionally, non-rural residents enrolled in Medicaid/Medicare had lower odds of lifetime suicide risk compared to those with other types of insurance (aOR = 0.78, 95% CI: 0.62–0.98).

### Non-Response Analysis Results

Given evidence suggesting that refusal or non-response to population-based suicide risk assessments may reflect an elevated risk of suicide,^40^, ^41^ a nonresponse analysis was conducted to explore the potential differences between respondents and non-respondents **(**Table 4). The results indicate that those who were not married or cohabitating (aOR = 0.60, 95% CI: 0.41–0.86) and those who reported fair or poor health (aOR = 0.63, 95% CI: 0.41–0.98) were significantly less likely to respond to the SBQ-R, when controlling for all other variables.

## DISCUSSION

To address the notable gap in research concerning lifetime suicide risk within rural areas of WV, a region affected by significant socioeconomic adversities, this study identified associations between socioeconomic and related factors and lifetime suicide risk while investigating rural and non-rural differences. Drawing from data obtained from the 2021–2022 MATCH survey, a state-based public health surveillance system, this study found that more than a quarter of WV adults indicated lifetime suicide risk, underscoring a significant prevalence of lifetime suicide risk in the state. Moreover, this study identified pertinent socioeconomic and related factors associated with lifetime suicide risk among WV adults living in rural and non-rural areas. Similarly to prior studies conducted outside of WV, our findings highlight the influence of socioeconomic and related factors such as marital status, education, poverty, health insurance coverage, health status, substance use, chronic pain, mental health disorders, and rurality on lifetime suicide risk.^4^, ^8^

Findings from our study suggest that rural residence may be a protective factor against lifetime suicide risk, though these results should be interpreted with caution. While previous research has suggested that rurality may be associated with increased lifetime suicide risk,^42^–^44^ our results suggest the opposite. Specifically, social connectedness has shown to have protective and buffering effects on lifetime suicide risk,^45^–^47^ and in rural communities, such as those found in WV, this connection may be fostered by shared values and a strong sense of belonging.^48^, ^49^ Additionally, the unique aspects of Appalachian culture and the strong influence of religion in WV may contribute to this protective effect.^50^–^52^ Appalachian culture emphasizes close-knit family ties, communal support, and resilience, while religious involvement provides social support, a sense of purpose, and coping mechanisms that buffer against lifetime suicide risk.^53^–^56^ However, it's important to consider that aspects of Appalachian culture, particularly stigma surrounding mental health, could act as a suppressive factor by discouraging individuals from acknowledging or reporting mental health struggles.^48^ In turn, this cultural stigma may offset the protective effects rurality seemingly had in this study. Furthermore, urban areas in WV may differ significantly from urban areas in other parts of the U.S., and this distinction could be influencing our results. Further research is necessary to determine whether these protective factors are unique to rural areas in WV or are influenced by other contextual factors.

Despite the potentially protective effects of rural residence and social connectedness against lifetime suicide risk, our results revealed that a commonly assessed indicator of social connectedness — marital status — did not have a significant association with lifetime suicide risk in rural areas. This finding is inconsistent with previous research that found increased odds of lifetime suicide risk among those who were not married or cohabitating.^20^, ^57^ However, measurement limitations in our study may have influenced these results. Marital status alone may not be the most effective indicator of social connectedness in this context, as it does not capture the quality or depth of relationships or the broader social environment. Additionally, challenges related to data collection, such as limited internet access and difficulties completing paper surveys, may have contributed to nonresponse bias, particularly in rural areas.^58^ These factors may have influenced the accuracy of marital status reporting and its association with social connectedness. Consequently, future research could benefit from exploring additional variables that may capture the nuances of social bonds in rural areas. Moreover, the protective effects of marriage on mental health and lifetime suicide risk are well documented in general suicide research, where marriage is often seen as providing emotional support, social stability, and economic security.^59^ However, there is limited research that has explored this in the Appalachian context. In the Appalachian context, other factors such as strong religious involvement and community ties may play a more significant role in buffering against lifetime suicide risk.^50^, ^51^ These cultural elements provide alternative forms of social support that might overshadow the protective effects typically associated with marital status. Furthermore, differences between Appalachian states and counties, such as varying levels of economic hardship, healthcare access, and community cohesion, may influence the relationship between marital status and lifetime suicide risk. WV, with its unique blend of strong religious presence and close-knit community values, may exhibit different patterns compared to other regions, highlighting the need for localized approaches to understanding and addressing lifetime suicide risk in Appalachia.

It is important to recognize, however, that while social connectedness may serve as a protective factor in Appalachian (and more broadly rural) communities, it is not sufficient on its own to address the broader issue of lifetime suicide risk. Bivariate results from our study indicate that WV adults living below the poverty threshold, those who were non-white, had less than a high school education, or were enrolled in Medicaid/Medicare had significantly greater odds of lifetime suicide risk. This highlights the critical role of socioeconomic deprivation in shaping vulnerability to suicide. These communities continue to face significant challenges, particularly socioeconomic deprivation, which includes limited access to mental health services, economic instability, and lower educational attainment. Additionally, high levels of stigma surrounding mental health further prevent individuals from seeking help, exacerbating feelings of isolation and hopelessness.^60^, ^61^ Economic hardship, in particular, has been shown to increase vulnerability to suicide, as poverty can intensify stress, limit access to healthcare, and restrict opportunities for social mobility. Therefore, interventions aimed at preventing suicide in rural areas must adopt a multifaceted approach, addressing both individual-level factors such as mental health and substance use, and community-level factors like economic development and improved access to services. Furthermore, it is crucial to recognize that variations in cultural, economic, and social contexts across different rural communities may result in differing risk factors and protective factors for suicide. This highlights the need for research that further explores these differences to develop tailored, localized intervention strategies.

For example, our study corroborates previous research on the intersectionality of chronic pain, mental health disorders, and lifetime suicide risk, particularly in communities with increased substance use, like WV.^10^, ^62^ Tailored suicide prevention efforts may consider incorporating chronic pain management, as chronic pain is particularly prominent in WV^63^ and has a strong association with increased lifetime suicide risk.^64^, ^65^ In this study, results showed that those with chronic pain, especially those residing in rural areas, had significantly greater odds of lifetime suicide risk. One explanation is that WV is predominantly a labor-intensive state, with a significant portion of the population employed in industries such as mining, logging, construction, farming, and manufacturing. These occupations involve strenuous manual labor, which contributes to workplace injury and the development of chronic pain over time.^66^ Similarly, heavy drinking has also been found to be heavily prevalent among those with chronic pain and these individuals are more likely to have mental health disorders like depression, anxiety, and PTSD,^67^ and heavy alcohol use has been found to compound these effects.^10^ Consequently, in the past, healthcare providers commonly prescribed opioids to labor workers for chronic pain management.^66^, ^68^ Sadly, this practice contributed to the over-prescription of opioids and exacerbated the current opioid crisis in WV.^68^ Individuals with chronic pain are more likely to have mental health disorders like depression, anxiety, and PTSD.^67^ Consequently, the elevated odds of lifetime suicide risk among WV adults reporting chronic pain, substance use, heavy drinking, and mental health disorders highlights the urgent need for integrated prevention and treatment approaches. The interplay of these factors not only compounds individual suffering but also exponentially increased the likelihood of suicide. This intricate web of issues demands immediate and comprehensive intervention.

By adopting a holistic approach that simultaneously tackles chronic pain management, substance use, mental health disorders, and heavy drinking, agencies can create more effective and cohesive treatment plans. Such strategies should involve multidisciplinary teams, including healthcare providers, mental health professionals, and substance abuse counselors, to ensure a comprehensive approach to each individual’s needs. Addressing these factors in a unified manner is crucial to mitigating their combined impact and improving outcomes for individuals at risk.^67^, ^69^ Interestingly, while lower levels of education have traditionally been associated with increased lifetime suicide risk,^70^ our multivariable analysis revealed a paradoxical finding: individuals with higher educational attainment in the WV population were more likely to report lifetime suicide risk. One plausible explanation could be that educational attainment has less variability across the state. For instance, the majority of adult WV residents have a high school diploma or equivalent, with a small percentage having less than a high school education or pursuing higher education.^71^ This demographic pattern suggests a reduced socioeconomic divide among WV residents, with a higher prevalence of individuals living in poverty.^20^, ^72^ Additionally, community-driven support networks in WV, such as mentoring programs and local educational initiatives, could help reduce educational disparities that may still exist by fostering strong social connections that provide crucial support to enhance educational opportunities within communities.^45^, ^47^, ^48^ The need to build these strong community networks through such initiatives aligns with the strain theory of suicide by serving as coping mechanisms that buffer against strain introduce by socioeconomic disparities. Furthermore, in areas like WV where lower educational attainment often correlates with high levels of religiosity,^73^ religion may act as an important protective factor not explored in this study. Approximately 79% of WV adults identify as Christian, and 22% consider themselves "very religious."^51^ This strong religious presence may provide additional layers of social and emotional support by strengthening an individual’s coping abilities and thereby potentially reducing suicide risk.^52^

### Implications of Findings

This study identifies the socioeconomic and related factors associated with lifetime suicide risk in rural WV; the implications of these findings can be used to inform suicide intervention efforts in rural WV. Our findings underscore the importance of addressing socioeconomic and related factors, such as substance use, chronic pain, mental health disorders, and access to healthcare in suicide prevention strategies tailored to the unique needs of WV communities.^74^–^76^ While our study suggests that individuals with higher education may exhibit a higher risk of suicide, this finding may reflect unique regional dynamics, suggesting that interventions should prioritize mental health services and social support rather than focusing on education alone. Specifically, prevention efforts should consider the unique cultural and socioeconomic characteristics of WV. Culturally sensitive interventions that leverage existing community resources and foster social cohesion could enhance resilience and reduce lifetime suicide risk among populations most at risk in WV.^77^

### Limitations

Despite the contributions of this study, several limitations should be acknowledged. First, the cross-sectional nature of the data precludes causal inference, and longitudinal studies are needed to clarify the temporal relationships between socioeconomic and related factors and lifetime suicide risk. Additionally, the cross-sectional design limits our ability to assess how SBQ-R scores correlate with subsequent suicide attempts or deaths. Therefore, future research should consider longitudinal approaches to evaluate the measure’s predictive validity. Second, while brief measures are often preferred in large-scale surveys to minimize respondent burden,^21^ research has shown that they can lead to misclassification and response bias.^78^ In our study, the percentage of missing responses to the SBQ-R was relatively low (3.24%). However, the non-response analysis revealed differences among respondents. Specifically, individuals who were not married or cohabitating and those who self-reported fair or poor health were less likely to respond to the SBQ-R, both of which have been associated with increased suicide risk.^79^–^82^ This result suggests that the non-responders may be at greater risk of suicide, which could potentially bias the results. Additionally, the SBQ-R measure for lifetime suicide risk complicates the interpretation of findings, as it is unclear whether socioeconomic variables contributed to lifetime suicide risk or if existing lifetime suicide risk contributed to these socioeconomic outcomes. Third, the rural population in our sample (39.79%) was lower than the reported state statistic of over 50%, possibly reflecting nonresponse bias among rural residents or differences in rurality classification using RUCC codes. However, results from the nonresponse analysis suggestion that rurality may not influence survey response rate. Fourth, data were collected between 2021–2022, during the COVID-19 pandemic. During this time, the prevalence of poor mental health was at an all-time high across the country and may have contributed to the high prevalence of lifetime suicide risk among our population.^83^ Fifth, lifetime suicide risk was assessed as a binary variable and did not explore the level of lifetime suicide risk. Sixth, the use of insurance status as a proxy for healthcare access may not fully capture the complexity of individuals' healthcare availability, access, or utilization, particularly in rural areas where proximity to services and provider shortages may be significant barriers. Furthermore, mental health measures for depression, anxiety, and PTSD were self-reported, which may introduce bias due to underreporting or misclassification of symptoms. Self-reported data can be influenced by stigma, recall bias, or individual interpretations of their mental health status, potentially limiting the accuracy of these measures. Future studies may benefit from incorporating objective health data or healthcare utilization records to better understand these relationships. Lastly, future research should examine predictors associated with lifetime suicide risk severity and qualitative approaches to potentially provide deeper insights into the underlying mechanisms driving lifetime suicide risk in rural WV.

## CONCLUSION

This study sheds light on the socioeconomic and related factors associated with lifetime suicide risk in WV, emphasizing the need for targeted interventions aimed at addressing the high prevalence of population lifetime suicide risk and enhancing community resilience. By elucidating the complex interplay between socioeconomic disparities in shaping lifetime suicide risk, our findings provide a foundation for evidence-based suicide prevention strategies tailored to the unique needs of WV communities. Moving forward, collaborative efforts between researchers, policymakers, and community stakeholders are essential for reducing suicide rates and promoting mental health and well-being in WV. Future research should expand to include other rural Appalachian areas to explore similarities or differences in community factors that may impact lifetime suicide risk. This broader approach will enhance our understanding and support the development of effective interventions across the region.

**SUMMARY BOX**

**What is already known about this topic?**

Socioeconomic deprivation, mental health disorders, substance use, and chronic pain are well-established risk factors for suicide. Rurality has been linked to both increased and decreased suicide risks, with prior studies highlighting the importance of cultural, social, and economic contexts. The role of these factors in shaping suicide risk in West Virginia (WV), a state characterized by substantial socioeconomic challenges and unique cultural dynamics, remains underexplored.

**What is added by this report?**

This study, using data from the 2021–2022 MATCH survey, highlights a significant prevalence of lifetime suicide risk among WV adults, with over a quarter reporting such risk. It identifies socioeconomic and related factors, such as chronic pain, substance use, poverty, and mental health disorders, as key contributors to suicide risk in WV. Notably, rural residence emerged as a potential protective factor, possibly due to social connectedness and cultural influences unique to Appalachia, though this finding contrasts with prior research.

**What are the implications for future research?**

Future research should explore the nuanced role of rurality as a protective factor against suicide risk, particularly in Appalachia. Longitudinal studies are needed to investigate the temporal dynamics of socioeconomic and related factors and their influence on suicide risk. Research should also focus on improving measurement tools for social connectedness and exploring alternative indicators, such as community cohesion and cultural factors. Tailored, localized interventions that address both individual and structural factors, such as chronic pain management, substance use treatment, and mental health services, are critical for suicide prevention in WV and similar Appalachian contexts.

## Figures and Tables

**Table 1:** Descriptive Statistics of West Virginia Adults With and Without Lifetime Suicide

	West Virginia		Lifetime Suicide Risk		No Lifetime Suicide Risk		
	freq.	%^b^	freq.	%	freq.	%	*p* a
**Overall**	15660	100.0%	3995	27.50%	11665	72.50%	
**Sex**							**0.03**
Male	5919	48.62%	1402	48.12%	4517	48.81%	
Female	9741	51.38%	2593	51.88%	7148	51.19%	
**Race**							**<.0001**
White	14210	93.62%	3583	92.30%	10627	94.12%	
Non-White	1400	7.63%	404	7.66%	996	5.67%	
Missing	50	0.16%	…	0.03%	42	0.21%	
**Married or Cohabitating**							**<.0001**
Yes	8047	54.29%	1823	45.70%	6224	57.55%	
No	7529	45.29%	2151	53.82%	5378	42.06%	
Missing	84	0.41%	21	0.48%	63	0.39%	
**Education**							
Less than HS	1590	11.97%	309	10.08%	1281	12.68%	**<.0001**
HS/GED or higher	13968	87.60%	3663	89.63%	10305	86.82%	
Missing	102	0.44%	23	0.29%	79	0.49%	
**Federal Poverty Level %**							
≤100%	4213	23.88%	1305	29.55%	2908	21.73%	**<.0001**
100.1%–199.9%	4068	23.39%	1084	25.96%	2984	22.41%	
≥200%	7379	52.73%	1605	44.47%	5773	55.86%	
Missing	…	00.01%	…	0.02%	…	0.00%	
**Insurance**							**0.001**
Medicaid/Medicare	6716	36.00%	1929	41.12%	4787	34.05%	
Other	7654	54.45%	1763	49.60%	5891	56.29%	
None	735	6.51%	203	6.09%	532	6.36%	
Missing	555	3.04%	100	2.37%	455	3.30%	
**Fair or Poor Health**							**<.0001**
Yes	4276	23.72%	1339	29.89%	2937	21.37%	
No	11384	75.99%	2656	69.83%	8728	78.32%	
Missing	63	3.00%	14	0.28%	49	0.31%	
**Substance Use**							**<.0001**
Substance use and heavy drinking	421	2.85%	200	4.94%	221	2.05%	
Substance use or heavy drinking	3984	26.83%	1573	40.39%	2411	21.69%	
No substance use or heavy drinking	11255	68.47%	2169	53.82%	8684	74.03%	**<.0001**
Missing	402	1.85%	53	0.85%	349	2.23%	
**Chronic Pain**							**<.0001**
Yes	4223	24.48%	1439	32.17%	2784	21.56%	
No	10341	70.15%	2389	64.56%	7952	72.27%	
Missing	1096	5.37%	167	3.27%	929	6.17%	
**Depression, Anxiety, or PTSD**							**<.0001**
Yes	3772	24.09%	1792	43.02%	1980	16.91%	
No	11846	75.72%	2193	56.79%	9653	82.91%	
Missing	42	0.19%	10	0.18%	32	0.19%	
**Rural Residence** b							**<.0001**
Yes	8372	39.79%	1990	36.62%	5283	59.01%	
No	7288	60.21%	2005	63.38%	6382	40.99%	
**Age**							**<.001**
18–34	2605	25.14%	1068	36.73%	1537	20.75%	
35–49	2852	22.21%	973	26.58%	1879	20.55%	
50–64	4354	27.56%	1084	24.24%	3270	28.81%	
65+	5738	24.49%	852	12.20%	4886	29.15%	
Missing	111	0.61%	18	0.25%	93	0.74%	
**Age ** ***(continuous)***	**Median**	**IQR**	**Median**	**IQR**	**Median IQR**	**<.0001**	
	50.17	30.02	40.99	26.08	53.72	29.60	

NOTE:

* Values less than 10 have been suppressed to follow data protection regulations

† High School/General Education Development (HS/GED); Post-Traumatic Stress Disorder (PTSD)

§ a P value for Rao Scott χ^2^

¶ b Percent represents weighted proportions

**Table 2:** Bivariate and Multivariable Associations Between Sociodemographic Characteristics and Suicide Risk

	Bivariate Results		Multivariable Results	
Variables	OR	95% CI	OR	95% CI
**Intercept**	---	---	0.69	0.54–0.87
**Age ** ***(continuous)***	**0.97**	**0.97–0.97**	**0.97**	**0.97–0.98**
**Sex**				
Male	0.97	0.87–1.09	1.07	0.94–1.21
Female	(ref)	(ref)	(ref)	(ref)
**Race**				
White	(ref)	(ref)	(ref)	(ref)
Non-White	**1.38**	**1.11–1.72**	1.04	0.82–1.33
**Married/Cohabitating**				
Yes	(ref)	(ref)	(ref)	(ref)
No	**1.61**	**1.44–1.80**	**1.35**	**1.19–1.54**
**Education**				
Less than HS	**1.30**	**1.07–1.58**	**0.61**	**0.49–0.76**
HS/GED or higher	(ref)	(ref)	(ref)	(ref)
**Federal Poverty Level %**				
≤100%	**1.71**	**1.50–1.95**	1.11	0.91–1.35
100.1%–199.9%	**1.46**	**1.27–1.67**	**1.23**	**1.03–1.47**
≥200%	(ref)	(ref)	(ref)	(ref)
**Insurance**				
Medicaid/Medicare	**1.37**	**1.22–1.54**	**0.80**	**0.67–0.94**
None	1.23	0.96–1.58	0.84	0.63–1.12
Other	(ref)	(ref)	(ref)	(ref)
**Fair or Poor Health**				
Yes	**1.57**	**1.39–1.77**	**1.51**	**1.29–1.77**
No	(ref)	(ref)	(ref)	(ref)
**Substance Use**				
Substance use and heavy drinking	**3.31**	**2.47–4.44**	**2.37**	**1.70–3.30**
Substance use or heavy drinking	**2.56**	**2.26–2.90**	**1.96**	**1.65–2.19**
No substance use or heavy drinking	(ref)	(ref)	(ref)	(ref)
**Chronic Pain**				
Yes	**1.67**	**1.48–1.89**	**1.40**	**1.20–1.64**
No	(ref)	(ref)	(ref)	(ref)
**Depression, Anxiety, or PTSD**				
Yes	**3.72**	**3.29–4.19**	**2.65**	**2.30–3.06**
No	(ref)	(ref)	(ref)	(ref)
**Rural Residence** a				
Yes	**0.83**	**0.75–0.93**	**0.87**	**0.77–0.98**
No	(ref)	(ref)	(ref)	(ref)

NOTE:

* High School/General Education Development (HS/GED); Post-Traumatic Stress Disorder (PTSD)

† Bolded estimates represent significant association with lifetime suicide risk; results are weighted

§ a Models adjusted for rural classification based on the 2023 Rural-Urban Continuum Codes (RUCC)

**Table 3:** Multivariable Logistic Regression Results Stratified by Rurality

Variables	Rural=1990		Non-Rural=2005	
	OR	95% CI	OR	95% CI
**Intercept**	**0.57**	**0.41–0.77**	**0.71**	**0.51–0.97**
Age (Continuous)	**0.98**	**0.97–0.98**	**0.97**	**0.97–0.98**
**Sex**				
Male	1.00	0.84–1.19	1.09	0.91–1.30
Female	(ref)	(ref)	(ref)	(ref)
**Race**				
White	(ref)	(ref)	(ref)	(ref)
Non-White	1.09	0.76–1.57	1.02	0.75–1.39
**Married or Cohabitating**				
Yes	(ref)	(ref)	(ref)	(ref)
No	1.13	0.95–1.35	**1.51**	**1.26–1.81**
**Education**				
Less than HS	**0.56**	**0.42–0.75**	**0.64**	**0.46–0.88**
HS/GED or higher	(ref)	(ref)	(ref)	(ref)
**Poverty**				
≤100%	1.14	0.88–1.48	1.11	0.84–1.47
100.1%–199.9%	1.19	0.95–1.48	1.25	0.97–1.60
≥200%	(ref)	(ref)	(ref)	(ref)
**Insurance**				
Medicaid/Medicare	0.85	0.68–1.07	**0.78**	**0.62–0.98**
None	0.86	0.57–1.28	0.80	0.55–1.81
Other	(ref)	(ref)	(ref)	(ref)
**Fair or Poor Health**				
Yes	**1.47**	**1.18–1.82**	**1.53**	**1.21–1.91**
No	(ref)	(ref)	(ref)	(ref)
**Substance Use**				
Substance use and heavy drinking	**3.75**	**2.24–6.28**	**1.75**	**1.44–2.12**
Substance use or heavy drinking	**2.23**	**1.85–2.71**	**1.74**	**1.15–2.62**
No substance use or heavy drinking	(ref)	(ref)	(ref)	(ref)
**Chronic Pain**				
Yes	**1.54**	**1.25–1.89**	**1.32**	**1.05–1.65**
No	(ref)	(ref)	(ref)	(ref)
**Depression, Anxiety, or PTSD**				
Yes	**2.25**	**1.85–2.73**	**2.93**	**2.41–3.57**
No	(ref)	(ref)	(ref)	(ref)

NOTE:

* High School/General Education Development (HS/GED); Post-Traumatic Stress Disorder (PTSD)

† Bolded estimates represent significant association with lifetime suicide risk; results are weighted

§ a Models adjusted for rural classification based on the 2023 Rural-Urban Continuum Codes (RUCC)

**Table 4:** Non-Response Analysis Results

	Multivariable	Non-Response Results
Variables	OR	95% CI
**Age** *(continuous)*	0.99	0.97–1.00
**Sex**		
Male	0.86	0.60–1.24
Female	(ref)	(ref)
**Race**		
White	(ref)	(ref)
Non-White	1.00	0.52–1.92
**Married/Cohabitating**		
Yes	(ref)	(ref)
No	**0.60**	**0.41–0.86**
**Education**		
Less than HS	0.63	0.38–1.03
HS/GED or higher	(ref)	(ref)
**Federal Poverty Level %**		
≤100%	0.98	0.58–1.67
100.1%–199.9%	1.29	.80–2.07
≥200%	(ref)	(ref)
**Insurance**		
Medicaid/Medicare	1.51	0.64–3.53
None	1.21	0.79–1.85
Other	(ref)	(ref)
**Fair or Poor Health**		
Yes	**0.63**	**0.41–0.98**
No	(ref)	(ref)
**Substance Use**		
Substance use and heavy drinking	1.12	0.75–1.67
Substance use or heavy drinking	1.74	0.67–4.52
No substance use or heavy drinking	(ref)	(ref)
**Chronic Pain**		
Yes	0.86	0.54–1.36
No	(ref)	(ref)
**Depression, Anxiety, or PTSD**		
Yes	0.69	0.46–1.06
No	(ref)	(ref)
**Rural Residence** a		
Yes	1.09	0.76–1.56
No	(ref)	(ref)

NOTE:

* High School/General Education Development (HS/GED); Post-Traumatic Stress Disorder (PTSD)

† Bolded estimates represent significant association with lifetime suicide risk; results are weighted

§ a Models adjusted for rural classification based on the 2023 Rural-Urban Continuum Codes (RUCC)
